# α ^+^-Thalassemia and Protection from Malaria

**DOI:** 10.1371/journal.pmed.0030221

**Published:** 2006-04-18

Over the course of human history, hundreds of thousands of genetic mutations have arisen in the global population. The most harmful ones usually disappear—by affecting an individual's “fitness,” i. e., the ability to reproduce, the mutations are lost before carriers can pass them on to their children—whereas most mutations are maintained in the population in low frequencies. Some mutations, however, can give the carrier such a large survival advantage that the mutations become positively selected for, leading to their presence in high frequencies in some populations.

Blood disorders are a good example of this selection process. The sickle cell mutation, for example, is a mutation of the β-globin gene that can cause severe anemia in people who inherit two mutated genes. People with just one mutated hemoglobin (Hb) S gene, however, can be highly protected against malaria. And in Africa, where malaria is one of the biggest killers, up to 40% of people are believed to carry one of the sickle genes.

The thalassemias are also inherited blood disorders that result from mutations in either the α-globin or β-globin genes. α-thalassemias are now the most common genetic disorders of human beings, and this is thought to be because of their protective effect against malaria. People usually have four α-globin genes, two on each Chromosome 16. In Africa, however, a common deletion can remove one of these genes from either chromosome or from both chromosomes; individuals with three or two α-globin genes remaining have anemia, which is more severe the fewer α-globin genes are present but is not life-threatening. This condition, in which at most one α-globin gene is missing from each chromosome, is known as α ^+^-thalassemia (α ^0^-thalassemia occurs when both genes are removed from a chromosome). Despite the well-known beneficial effect of α ^+^-thalassemia against malaria, scientists know little about how exactly the α ^+^-thalassemias result in this protection, and whether they protect against all forms of the disease.

In a new study in *PLoS Medicine*, Thomas Williams, Sammy Wambua, and colleagues—researchers from Kenya and Oxford—investigated the effect of α ^+^-thalassemia on malaria and other childhood diseases, such as gastroenteritis, in two groups of children in Kenya. The first group comprised children younger than five years old, recruited between September 1998 and August 2001, 301 of whom were analyzed in the study (the mild disease cohort). The second group comprised 2,104 children recruited at birth between May 1992 and April 1995 (the birth cohort). All children analyzed were typed for both HbS and α ^+^-thalassemia.

Williams and colleagues found that α ^+^-thalassemia (either with one or two α-globin genes lost) was associated with significant reductions in the rate of admission to hospital with malaria (with or without signs of severity) and severe malaria. Both homozygous individuals (with two α genes missing in total, one from each chromosome) and heterozygote individuals (with only one α gene missing in total) had much lower rates of severe malaria anemia than normal children.

However, α ^+^-thalassemia had no effect on symptomless parasitemia (defined as the presence of the *Plasmodium falciparum* malaria parasite in the blood of a child, but without fever or other symptoms). And although the occurrence of uncomplicated malaria was lower in both those heterozygous and those homozygous for α ^+^-thalassemia compared with normal children, this drop in incidence was not statistically significant.

In general, there were no links between α ^+^-thalassemia and the occurrence of nonmalarial illnesses. There were, however, two exceptions. In the birth cohort, fewer heterozygous infants than normal children were admitted to hospital with severe anemia. In the mild disease cohort, both heterozygous and homozygous children had a lower frequency of lower respiratory tract infections than normal children—although, this was not seen in the other cohort.

These findings appear to contradict previous results from a study conducted by the same group in the Pacific islands of Vanuatu that showed α ^+^-thalassemia might increase the frequency of uncomplicated malaria. The authors say this might partly be explained by the fact that, unlike Kenya, in Vanuatu two species of malaria, *P. vivax* and *P. falciparum*, both cause disease at similar frequencies. Furthermore, there may be major genetic differences in both human and parasite populations between these regions.

Whether the mutation has a protective effect against other diseases is still unresolved. The authors maintain that such an effect is plausible—indeed, the protection from lower respiratory tract infections in some children was of a similar magnitude to that for malaria. The authors have also previously recorded protection against nonmalarial diseases in Papua New Guinea.

These findings have important implications for researchers looking for “antimalarial” genes and for those searching for potential vaccine candidates: they will need to use a carefully focused approach that differentiates between uncomplicated, or symptomless, disease and the complicated, or severe, form.

